# Estimating the economic burden of mastitis and antimicrobial use in a commercial dairy system using the animal health loss envelope framework

**DOI:** 10.3389/fvets.2026.1763368

**Published:** 2026-04-07

**Authors:** Chisoni Mumba, Wizaso Mwasinga, Ntombi Mudenda, Anafi Mataka, Mohamed Moctar Mouliom Mouiche

**Affiliations:** 1 Department of Disease Control, School of Veterinary Medicine, University of Zambia, Lusaka, Zambia; 2 Department of Clinical Studies, School of Veterinary Medicine, University of Zambia, Lusaka, Zambia; 3 African Society for Laboratory Medicine, Addis Ababa, Ethiopia

**Keywords:** AHLE framework, AMU, dairy cattle, economic burden, mastitis, Zambia

## Abstract

**Introduction:**

Mastitis is one of the most economically important diseases affecting dairy production systems, generating substantial losses through reduced milk production, treatment costs, and premature animal removal. However, farm-level economic assessments based on longitudinal management data remain limited in many low- and middle-income countries.

**Methods:**

This study applied the Animal Health Loss Envelope (AHLE) framework to estimate the economic impact of mastitis using four years (2022–2025) of routinely collected data from a large commercial dairy farm in Zambia. Farm records were analyzed to quantify disease occurrence, treatment outcomes, antimicrobial use, production losses, and health-related expenditures associated with mastitis management.

**Results:**

The herd recorded an average of 768 clinical mastitis cases annually among approximately 1,809 lactating cows, corresponding to an average incidence of 42.46%. Although recovery rates were high (95.1%), mastitis resulted in substantial productivity losses through discarded milk, mortality, and mastitis-related culling. Economic impacts were driven by both direct expenditures on treatment and preventive interventions, including antimicrobial therapies, diagnostics, hygiene measures, and vaccination, as well as indirect losses associated with reduced marketable milk and animal removal. Antimicrobial use intensity (AMUI) showed temporal variation across production periods, with higher AMUI values observed during months with increased mastitis incidence. Annual antimicrobial expenditure exceeded ZMW 834,833.00 (USD 34,784.70) while AMR-related diagnostics and mitigation investments (vaccination, disinfectants, biosecurity) exceeded ZMW 2,977,667.00 (USD 193,638.80). Mastitis caused large indirect losses through discarded milk (up to 234,445.25 L/year) and delayed reproduction (395-day calving interval). The final estimated loss of ZMW 6,786,211.88 (USD 282,758.83) resulted from the combined effects of direct disease management costs and indirect productivity losses observed per year.

**Conclusion:**

The results illustrate how mastitis and treatment-refractory infections contribute significantly to the Animal Health Loss Envelope (AHLE) and provide foundational data for future Productivity-adjusted-life-years (PALYs) estimations and cost-effectiveness analyses of AMR mitigation strategies. Our study demonstrates the practical applicability of the GBADs AHLE framework in real-world dairy systems.

## 1 Introduction

Mastitis is widely recognized as one of the most economically important diseases affecting dairy production systems worldwide (1). The disease imposes substantial losses through reduced milk yield, discarded milk, increased veterinary and treatment expenditures, reproductive inefficiencies, pre-mature culling, and animal mortality (1, 2). Previous studies have demonstrated that the economic consequences of mastitis vary considerably across production systems, reflecting differences in pathogen profiles, management practices, diagnostic approaches, and local economic conditions (1, 2). Estimates from international studies indicate that mastitis can generate substantial financial losses at both herd and industry levels, emphasizing the importance of understanding its economic implications within specific production contexts (1).

In addition to direct production losses, mastitis influences broader farm management decisions, including treatment strategies, labor allocation, biosecurity investments, and herd replacement planning. These impacts extend beyond individual clinical events and may influence overall farm productivity and long-term profitability (2). As dairy production intensifies in many low- and middle-income countries, particularly in sub-Saharan Africa, the economic burden associated with mastitis is expected to become increasingly important. However, empirical farm-level economic assessments based on detailed longitudinal data remain limited in these regions, largely due to challenges in accessing reliable production and management records from commercial farms (3, 4).

Quantifying the economic impact of livestock diseases requires analytical approaches capable of integrating multiple dimensions of loss, including morbidity, mortality, and health-related expenditures. The Animal Health Loss Envelope (AHLE), developed under the Global Burden of Animal Diseases (GBADs) programme, provides a structured framework for estimating disease-related losses by combining production impacts with costs associated with disease prevention, treatment, and control (5). By capturing both productivity losses and health expenditures, the AHLE framework offers a comprehensive approach for assessing the economic consequences of animal diseases using routinely collected farm-level data (5).

Mastitis management typically involves a combination of preventive measures, supportive care, and antimicrobial treatment, all of which contribute to farm-level disease-related expenditure. Antimicrobials are widely used in livestock systems to reduce infectious disease losses and support animal health, although their use also raises broader considerations regarding antimicrobial stewardship and sustainability (6, 7). While antimicrobial use represents an important component of mastitis management, the primary focus of this study is to quantify the overall economic impact of mastitis at farm level rather than to estimate pathogen-level antimicrobial resistance burden. Understanding the full economic consequences of mastitis remains an essential step for informing evidence-based disease control strategies and improving resource allocation in dairy production systems (2).

Therefore, this study applies the Animal Health Loss Envelope framework to estimate the economic impact of mastitis using 4 years (2022–2025) of routinely collected management and production data from a large commercial dairy farm in Zambia. In addition to estimating the economic burden of mastitis, the study evaluates antimicrobial use intensity (AMUI) to characterize patterns of antimicrobial consumption within farm-level mastitis management. The study provides empirical evidence on the scale of mastitis-related economic losses within an intensive dairy production system and demonstrates the practical application of the AHLE framework for farm-level disease burden assessment by integrating disease occurrence, treatment expenditure, production losses, and herd outcomes.

## 2 Materials and methods

Ethical consideration: ethical approval for this study was obtained from the Excellence in Research Ethics and Science (ERES) Converge Zambia Institutional Review Board (Reference number: 2024-Dec-023), which approved the broader research project under which this farm-level analysis was conducted. The approval covered the use of retrospective farm records, antimicrobial use data, production records, and animal health information.

This study did not involve any experimental manipulation, invasive procedures, or direct handling of animals by the research team. All data analyzed were routinely collected by the farm as part of standard herd health, mastitis management, and production monitoring activities. Permission to access and use these records was granted by the farm management. All farm-level data were anonymised prior to analysis to ensure confidentiality.

Because the study relied exclusively on secondary data and involved no modification of veterinary treatment or farm practices, it posed no risk to animals or personnel. The research team adhered to ethical principles of data integrity, confidentiality, and responsible reporting, consistent with the University of Zambia's research ethics guidelines.

### 2.1 Study design

A retrospective analysis was conducted using 4 years (2022–2025) of routinely collected farm data from a large commercial dairy farm in Zambia. The study used the AHLE Conceptual Framework to classify and quantify mastitis-associated losses into: production losses due to mortality, Production losses due to morbidity, Health expenditures, including AMU and AMR mitigation, and Negative externality pathways, including treatment failure and prolonged disease (1).

### 2.2 Data sources

Data for this analysis were drawn from comprehensive farm-level records (called Mastitis Monitoring) routinely maintained by the commercial dairy enterprise between 2022 and 2025. These records covered all components required to apply the GBADs AHLE conceptual framework, including disease incidence, treatment practices, expenditure, and production parameters as shown in Table 1. Specifically, mastitis occurrence data were obtained from daily health registers that documented new clinical and subclinical cases, recurrence, severity classification, clinical progression, recovery, mortality, and decisions for culling. The farm classified mastitis cases as mild (abnormal milk only without udder swelling or systemic signs), moderate (abnormal milk with udder inflammation but no systemic illness), and severe (abnormal milk with udder inflammation and systemic illness), and recorded the affected udder quarter(s) and the corresponding severity score for each case. Mortality logs were used to identify deaths directly attributable to mastitis-associated complications such as toxemia and gangrenous mastitis. Culling records provided information on animals removed due to chronic mastitis, irreversible udder damage, or treatment failure.

**Table 1:** Data needs for the AHLE framework.

Data category	What's needed
Antimicrobial use (AMU) data	- Data on antimicrobial types and quantities used at the farm. - Retail price of antimicrobials used.
AMR frequency measures	- Incidence and prevalence rates of AMR-mastitis.
Production losses due to mortality	- Mortality rates linked to AMR mastitis infections. - Animal disposal costs due to AMR-mastitis.
Production losses due to morbidity	- AMR-related reductions in production efficiency, including: - Delayed selling or product withdrawal. - Increased pre-mature culling and replacement costs. - Milk Yield reductions. - Reproductive impacts (reduce calving intervals).
Health expenditure	Costs of treating AMR-infected livestock, including: - Additional or second-line therapies. - More expensive diagnostic tests (AST). - Veterinary services and farm labor. - AMR prevention and mitigation (e.g., biosecurity, vaccination, outbreak control).

Milk production data were sourced from electronic milking parlor systems, which captured daily individual cow yields. This allowed quantification of both discarded milk during mastitis episodes and contaminated milk diverted to calves during treatment and withdrawal periods.

Reproductive performance indicators, including calving interval and reproductive efficiency metrics routinely recorded by the farm, were used to evaluate indirect productivity effects occurring during the study period. These parameters were analyzed at herd level rather than being causally linked to individual mastitis cases.

No cow-level causal modeling was performed to directly attribute reproductive changes to mastitis episodes. Instead, reproductive impacts were interpreted as part of the broader herd-level productivity context in which mastitis occurred, consistent with the Animal Health Loss Envelope (AHLE) framework that incorporates indirect productivity losses associated with disease.

Antimicrobial use data were obtained from pharmacy purchase records and treatment logs. These included the type and quantity of intramammary tubes administered, systemic antibiotics used, supportive therapies dispensed, and dosages per treatment course. Unit prices were extracted from farm invoices to estimate annual health expenditure attributable to mastitis. Additional data on preventive interventions, such as disinfectants, sanitation chemicals, farm biosecurity inputs, and mastitis vaccination, were obtained from procurement ledgers and application logs.

Diagnostic data were sourced from laboratory invoices and diagnostic reports documenting the number of milk samples submitted monthly for bacterial culture and antimicrobial susceptibility testing (AST). These reports also provided pricing information for laboratory services, enabling estimation of AMR-related diagnostic expenditure. Together, these diverse datasets provided a comprehensive empirical basis for mapping mastitis-associated losses into the production and expenditure pathways defined by the AHLE framework.

### 2.3 Operationalization of the animal health loss envelope framework

This study applied the Animal Health Loss Envelope (AHLE) framework to quantify the economic burden associated with mastitis at farm level. The AHLE framework provides a structured approach for integrating disease occurrence, production losses, and health-related expenditures to estimate the overall impact of livestock diseases within production systems.

Operationalisation of the framework was undertaken by mapping routinely collected farm data to key AHLE components show in Table 1. Disease occurrence was represented by annual clinical mastitis cases, treatment outcomes, mortality, and mastitis-related culling obtained from farm health records. Production losses were quantified using indicators such as discarded milk and changes in productivity-related parameters recorded during the study period. Health expenditures included treatment costs, antimicrobial use, diagnostics, and preventive interventions implemented as part of mastitis management.

Within this framework, antimicrobial use intensity (AMUI) was included as a management indicator describing the intensity of antimicrobial consumption associated with mastitis treatment. AMUI was used to characterize antimicrobial use patterns in relation to disease occurrence and management practices rather than as a direct measure of antimicrobial resistance.

The AHLE framework enabled the estimation of direct and indirect economic pathways through which mastitis affected herd productivity and farm profitability by integrating these epidemiological, production, and economic data streams.

### 2.4 Analytical approach

We started with profiling the herd structure for the farm over a period of 4 years (2022–2025). It is from the herd structure that we estimated the incidence rate of mastitis at the farm. The estimation of the incidence rate was based monthly incidence of clinical mastitis per year by dividing the number of cases by the number of lactating cows multiplied by 100. We then obtained averages for each year from 2022–2024 to come up with an average for the 4 years.

Then we mapped antimicrobial use (AMU) to the farm's health expenditure accounts. All intramammary and systemic antimicrobials used for mastitis treatment were treated as direct expenditures incurred to reduce infection-related losses. Supportive therapies, labor associated with treatment, and diagnostic procedures were similarly categorized as health expenditures aligned with the AHLE framework.

The second component of the analysis focused on quantifying production losses attributable to morbidity. Using milk yield data, we calculated the total volume of milk discarded during treatment and withdrawal periods, as well as milk downgraded to calf feeding. These losses represent reductions in marketable output and were therefore treated as morbidity-related productivity losses within the AHLE.

Mortality and culling represented the third analytical pathway. Deaths from severe mastitis were categorized as direct production losses due to mortality, capturing the loss of future milk output, reproductive potential, and the costs of carcass disposal. Pre-mature culling due to chronic or treatment-refractory mastitis was also analyzed under production losses, reflecting foregone lifetime productivity and replacement costs for newly introduced heifers.

The fourth analytical dimension addressed AMR-specific impacts. Treatment failure, evidenced by prolonged clinical courses, need for second-line antibiotic regimens, or culling following unresponsiveness, was interpreted as a manifestation of possible antimicrobial resistance. Additional expenditures incurred in such cases, including repeated treatments and more expensive therapeutic alternatives, were mapped to the AMU/AMR-associated health expenditure pathways outlined in the AHLE framework. The cost of antimicrobial susceptibility testing was considered a direct AMR expenditure, justified by the need to confirm pathogen susceptibility patterns and guide therapeutic decision-making.

Expenditures on preventive AMR mitigation, such as vaccination against mastitis, disinfectants, biosecurity inputs, and on-farm hygiene enhancement, were incorporated into the analysis as proactive investments aimed at reducing future AMR-related burdens.

**Figure 1:**
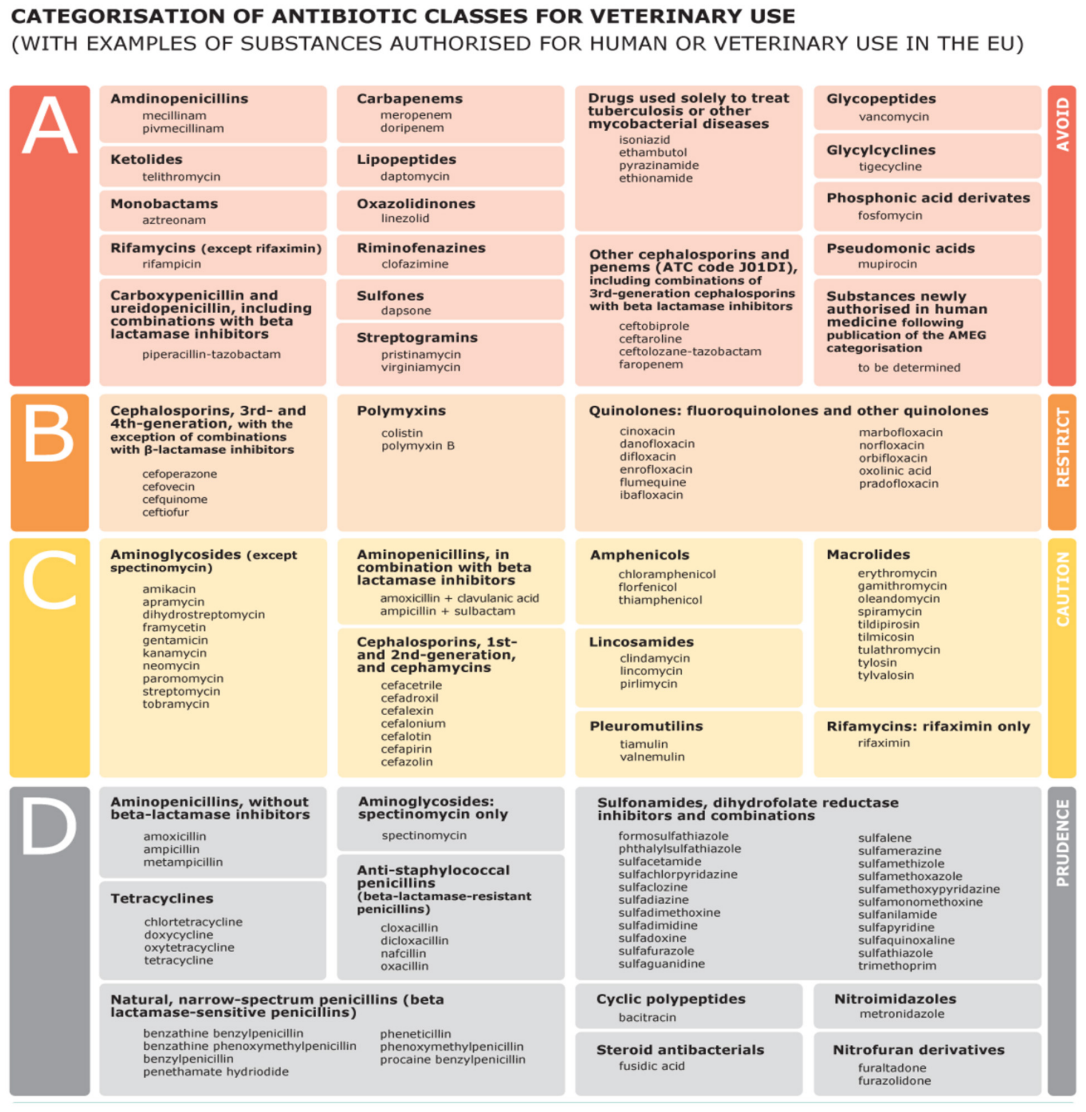
Classification of antimicrobials (8).

Antimicrobial Use Intensity (AMUI) data were compiled from batch-level farm treatment and procurement records covering January–December each of the 4 years. For each monthly batch, antimicrobials were grouped according to Class A–D categories (Figure 1) based on European Medicines Agency (8), and total antimicrobial product used (mg) was calculated. Animal biomass produced (kg) per batch was extracted from production logs, where each lactating cow was routinely weighed during milking sessions. Using biomass as the denominator allows antimicrobial use to be standardized relative to the animal population at risk and is consistent with international approaches for reporting antimicrobial use in livestock (9).

Antimicrobial Use Intensity (AMUI) was computed using the formula:

*AMUI (mg/kg)* = *total antimicrobial product used (mg)* ÷ *total animal biomass produced (kg)* *(**9**)*.

This allowed standardized comparison of AMU across batches and seasons.

## 3 Results

### 3.1 Herd structure, production characteristics and mastitis occurrence

Across the 4-year study period (2022–2025), the number of lactating cows ranged from 1,738 to 1,902, with an average of 1,809 animals per year as shown in Table 2. The herd consisted Holstein-Friesian breeds managed under intensive system with twice-daily milking (a.m. and p.m.) and an average herd milk yield of 30.12 L per cow per day. Voluntary culling for poor production was implemented when milk yield fell below 15 L per day, particularly when combined with poor reproductive performance.

**Table 2:** Herd-level mastitis occurrence, treatment outcomes, and removal indicators used for economic impact analysis (2022–2025).

Year	Lactating cows	Clinical mastitis cases	Mortality	Cows treated	Cows recovered	Cows culled	Mastitis incidence (%)	Recovery rate (%)	Culling rate (%)
2022	1,738	729	13	729	678	38	41.94	93.0	2.19
2023	1,843	888	18	888	849	21	48.18	95.6	1.14
2024	1,751	722	8	722	683	31	41.23	94.6	1.77
2025	1,902	732	12	732	711	9	38.49	97.1	0.47
Average	1,809	768	13	768	730	25	42.46	95.1	1.39

Lactation management followed a structured schedule: heifers were first served at 13 months, entered first lactation at 24 months (2 years), were inseminated 60 days post-calving, and then dried off at 7 months. Cows typically entered second lactation at 3–4 years of age, and culling was instituted after five failed inseminations. Some cows remained in production for up to nine or 10 lactations, although mastitis was most frequently observed in the third lactation, consistent with cumulative udder damage and reduced immune resilience.

Mastitis occurred throughout the year but peaked during periods of high milk production (June) and the rainy season (December–January).

Across the 4-year period (2022–2025), clinical mastitis represented a substantial health burden, with annual case numbers ranging from 722 to 888 cases and an overall average of 768 cases per year. No subclinical mastitis cases were formally recorded within the farm database during the study period. Subclinical mastitis was assessed using a field-based screening approach (California Mastitis Test) based on milk consistency changes. Cases were classified as trace (+1) when a slight slime was observed that disappeared quickly, mild inflammation (++2) when distinct slime was present without gel formation, and moderate inflammation (+++3) when immediate thickening with slight gel formation was observed.

In low-yielding cows, most subclinical cases were classified as trace (+1) or mild (++2). These animals were managed conservatively using supportive therapy rather than antimicrobial treatment. Supportive management included administration of dairy drench, vitamin B complex, ketoprofen, and oxytocin in selected cases exhibiting incomplete milking. Animals were subsequently monitored for approximately 2 days to assess clinical progression and determine whether further intervention was required. Much as subclinical mastitis cases were not formally recorded in the farm database, the cost of subclinical mastitis was covered under supportive treatment.

Clinical Mastitis incidence remained consistently high, varying between 38.49 and 48.18%, with a mean incidence of 42.46%. Treatment coverage corresponded closely with reported clinical cases, indicating that all affected animals received intervention. Recovery rates were consistently high across years, ranging from 93.0 to 97.1%, with an overall mean of 95.1%, suggesting effective clinical management despite sustained disease pressure.

Mortality associated with mastitis ranged from eight to 18 animals annually, while mastitis-related culling varied from nine to 38 cows per year, corresponding to an average culling rate of 1.39%. Although recovery rates were high, mortality and culling represented permanent losses of productive animals and therefore constituted important contributors to the overall economic impact of mastitis within the herd, as captured by the AHLE framework.

### 3.2 Clinical course and removal of affected cows

On the farm, the average treatment duration for mastitis was 2 days, with the severity of production loss ranging from mild to severe depending on the severity of the case and timeliness of intervention. Severe cases were reported to often require additional therapy based on the presence of systemic signs such as diarrhea, dehydration, fever and lethargy which may prolong recovery time. On average, 25 cows were culled annually for mastitis compared to 13 deaths, underlining the role of mastitis as a driver of pre-mature exit from the herd, as shown in Table 2.

Cows that succumbed to mastitis were disposed of by incineration, while those failing to respond to treatment after 8 days were culled and slaughtered at the abattoir, with carcasses entering the beef value chain after withdrawal periods. This removal pathway has implications for animal welfare, biosecurity, and economic returns from salvage slaughter. The price of incineration could not be established because the dairy farm is integrated with the beef enterprise, thus once the animal leaves the farm for incineration or culling, it is no longer part of dairy enterprise.

### 3.3 Antimicrobial use patterns for mastitis treatment

Mastitis therapy at the farm relied on a combination of intramammary and systemic antimicrobial regimens, supported by adjunctive therapies. The first-line intramammary products used were: Cephalexin 200 mg + Kanamycin 100,000 IU, and Tetracycline + Neomycin + Bacitracin + Prednisolone. Systemic therapy included injectable preparations such as: Amoxicillin + Clavulanic acid, and Trimethoprim + Sulfadoxine.

Supportive therapy was provided using Drench (calcium propionate, yeast, minerals, niacin) to restore electrolyte balance and support recovery.

Treatment protocols stipulated that affected quarters receive intramammary therapy for 2–3 days; if no response was observed, a second-line intramammary product was used for an additional 4 days. Milk from treated cows was discarded for 6 days (2 days treatment + 4 days withdrawal) and, when appropriate, antibiotic-contaminated milk was fed to calves, which is a driver of antimicrobial resistance.

Across the 4-year period, treatment success was generally high, with recorded clinical cure rates of 93.0% (2022), 95.6% (2023), 94.6% (2024) and 97.1% (2025), indicating persistent treatment failures and potential antimicrobial resistance in a minority of cases based on Antimicrobial Susceptibility Testing (AST).

### 3.4 Milk loss and impact on reproductive performance

Milk productivity losses constituted a major component of the economic burden associated with mastitis. During the study period, substantial volumes of milk containing antimicrobial residues were either discarded or diverted from sale due to treatment and withdrawal requirements. Total discarded milk amounted to 937,781 liters, corresponding to an annual average of 234,445 liters as shown in Table 3.

**Table 3:** Volume of discared milk due to antibiotic treatment of mastitis.

Year	Dicarded milk (with antibiotic residues) in liters	Milk fed to calves (with antibiotic residues) in liters
2025	177,219	63,689.2
2024	210,899	76,135
2023	298,710	33,148.8
2022	250,953	22,950
Total	937,781.00	195,923.00
Average	234,445.25	48,980.75

In addition, a total of 195,923 liters of milk containing antimicrobial residues was fed to calves, representing an average of 48,981 liters annually. Although this milk was retained within the production system, it represented a loss of marketable product and therefore reduced potential revenue from milk sales.

Year-to-year variation in milk losses reflected fluctuations in mastitis incidence and treatment intensity, illustrating the linkage between disease occurrence, management responses, and productivity losses. The price of milk per liter was K13.5 multiplied by an average amount of milk discarded (234,445 L) due to mastitis resulted in a loss of ZMW 3,165,010.88 (USD 131,875.5) per year as shown in Table 4. Within the AHLE framework, these outcomes represent morbidity-related production losses that substantially contributed to the overall economic burden observed at herd level.

**Table 4:** Direct and indirect costs of treating mastitis.

Anti microbials	Unit	Unit price (K)	Use month	Use/year	Cost per year (ZMW)	Cost/year (USD)
Intramammary						
Cephalexin B.P 200 mg + Kanamycin Suplhate BP 100,000 I.U one tube per affected quarter twice a day	Tube	79.25	280	3,360	266,280.00	11,095.00
15.6-7.5,-14.1499ptTetracycline 200 mg + Neomycin 250 mg + Bacitracin 2,000 IU + Prednisolone 10 mg two tubes per affected quarter twice a day	Tube	106.55	82	984	104,845.00	4,368.55
Injectables						
Amoxicillin 140 mg/ml + Clavulanic acid 35 mg/ml 8.75 mg/kg IM SID for 3–5 days	100 ml	1,101.73	10	120	132,208.00	5,508.65
15.6-7.5,-14.1499ptTrimethoprim + Sulfadoxine 240 mg/ml 16 mg/kg every 24 h IV/IM for 3–5 days	100 ml	160	25	300	48,000.00	2,000.00
Drench-mate drench						
Calcium propionate + yeast + minerals, niacin + other nutrients 680 g in 25 L warm water once a day for 2 days	22.72 kg	6,750	3.5	42	283,500.00	11,812.50
Subtotal					834,833.00	34,784.70
Prevention and control						
Chlorine alkali milk pipelines during C.I.P cleaning process	200 L	9,664	2	24	231,936.00	9,664.00
Chlorine surface of the calf shed	25 kg	1,504	4	48	72,192.00	3,008.00
Alkaline detergent Equipment cleaning the milking yparlor	25 L	1,262	14	168	212,016.00	8,834.00
Cypermethrin + cymiazol	20 L	12,013.7		28	336,384.00	14,016.00
Subtotal of cost of prevention and control					852,528.00	35,522.10
Diagnostics						
Antimicrobial susceptibility testing		188	15	12	33,840.00	1,410.00
Vaccine						
Vaccine against *Staphylococcus aureus*, coliforms and coagulase-negative staphylococci vaccinated at 52 DIM, 45 days before calving and 10 days before calving five doses	Five doses (10 ml)	508.09	343	4,116	2,091,298.00	87,137.44
Subtotal					2,977,667	193,638.8
Volume of milk discarded (average) and number of cows culled or died						
Milk	L	13.5		234,445	3,165,010.88	131,875.5
Cows culled and died	Cows	50,000		38	1,900,000	75,000.00
Subtotal					5,065,010.88	211,042.1
Grand total					6,786,211.88	282,758.83

Exchange rate of USD 1.00 = ZMW 24.00 (Bank of Zambia 2025).

In addition to direct milk loss, mastitis prolonged the calving interval by approximately 15–20 days, resulting in an average calving interval of 395 days. This extension reflects systemic illness, reduced feed intake and hormonal disturbances linked to infection and treatment. Longer calving intervals reduced annual milk yield per cow, increased insemination costs, lengthened non-productive periods, and ultimately lowered lifetime productivity. However, these reproductive inefficiencies were not captured in the economic cost.

### 3.5 Economic burden of treating drug-resistant mastitis

Drug-resistant or treatment-refractory mastitis cases required more intensive and prolonged therapy, including combined intramammary and systemic antibiotics, NSAIDs or corticosteroids, and fluid/electrolyte support. Cases diagnosed as gangrenous mastitis were considered untreatable and culled.

The annual cost of treating mastitis is summarized in Table 4. Together, these expenditures demonstrate that drug-resistant and severe mastitis impose a substantial, recurrent annual cost on the farm, above and beyond the indirect losses from reduced milk yield, fertility and culling.

In addition, culture and antimicrobial susceptibility testing (AST) were performed to guide therapy in selected cases. An average of 15 milk samples per month were submitted to a private laboratory at a cost of ZMW 188.09 per sample, resulting in an annual AST expenditure of ZMW 33,856.20 (USD 1,410.00).

### 3.6 Costs of AMR prevention and control measures

The farm invested in preventive AMR control strategies, including disinfection, vaccination, targeted antibiotic use and surveillance. Routine hygiene measures relied on multiple disinfectants: chlorine alkali for cleaning-In-Place of milk pipelines, chlorine-based disinfectant for calf sheds, alkaline detergent for milking parlor equipment, and Cypermethrin + Cymiazol for ectoparasite control and housing disinfection.

Based on usage patterns, the total annual cost for disinfection was ZMW 852,528.00 (USD 35,522.10). In addition, a vaccine, targeting *Staphylococcus aureus*, coliforms and coagulase-negative staphylococci, was administered according to a three-dose schedule (around 52 days in milk and pre-calving). With an estimated 4,116 doses per year at ZMW508.09 (USD 21.17) per 5-dose unit, the annual vaccination cost was approximately ZMW 2,091,298 (87,137.44) as show in Table 4. The farm complemented these measures with:

- Selective Dry Cow Therapy (SDCT) to reduce unnecessary antimicrobial exposure,
- On-farm culture and sensitivity testing to target treatment and avoid broad-spectrum drugs,
- Farm-level AMR surveillance through periodic bacterial culture and resistance profiling,
- AMU audits and veterinary oversight, including restricted use or banning of fluoroquinolones and 3 rd/4 th generation cephalosporins (e.g. Cobactan and Marbocyl banned since 2023.

### 3.7 Integrated interpretation of economic losses using the AHLE framework

Application of the Animal Health Loss Envelope (AHLE) framework enabled the economic burden of mastitis to be interpreted through interconnected disease, management, and productivity pathways. The final estimated loss of ZMW 6,786,211.88 (USD 282,758.83) resulted from the combined effects of direct disease management costs and indirect productivity losses observed per year (Table 4).

Within the AHLE structure, disease occurrence represented the primary driver of losses, as reflected by the sustained mastitis incidence recorded across years. High disease burden required continuous treatment interventions, including antimicrobial therapy and supportive care, which contributed substantially to direct health expenditures. Prevention and control investments, including hygiene management and vaccination, represented additional costs incurred to reduce disease recurrence and maintain herd health performance.

The largest proportion of total losses arose from productivity-related components, particularly discarded milk and animal removal through culling or mortality. These findings highlight that the economic burden was shaped more by downstream production impacts than by treatment expenditure alone. In this context, antimicrobial use intensity (AMUI) reflected the level of management response required to control disease pressure, linking clinical burden to resource utilization within the production system.

Overall, the AHLE framework demonstrated that the economic impact of mastitis emerged through a disease-management-productivity pathway in which persistent mastitis incidence increased treatment intensity and antimicrobial use, leading to both direct expenditures and indirect losses in production efficiency. This integrated interpretation illustrates how routine farm-level data can be organized within a structured economic framework to capture the full envelope of livestock disease losses.

### 3.8 Antimicrobial use intensity

Batch-level AMU results revealed substantial seasonal and class-specific variation. Classes C and D (Figure 1) accounted for the highest antimicrobial quantities used, with AMUI values ranging from as low as 0.03 mg/kg (Batch 2, June 2024) to as high as 4.64 mg/kg (Batch 1, January 2024). Biomass ranged between 1.71 million and 2.01 million kg across months. Higher AMU levels were recorded during January–March and again in November–December, aligning with peak mastitis periods on the farm as shown in Table 5.

**Table 5:** Antimicrobial use intensity at a commercial dairy farm.

Batch number and date	Antimicrobial class	Total antimicrobial used (mg)	Total animal biomas	AMUI (mg/Kg)
Batch 1: 1/01/2024 TO 31/01/2024	Class A	0		0
	Class B	448,000	1,835,386	0.24
	Class C	8,511,600		4.64
	Class D	2,340,680		1.28
Batch 2: 01/02/2024 to 29/02/2024	Class A	0	1,835,386	0
	Class B	420,000		0.23
	Class C	3,332,400		1.82
	Class D	3,945,700		2.15
Batch 3: 01/03/2024 to 31/03/2024	Class A	0	2,012,643	0
	Class B	285,000		0.14
	Class C	3,756,500		1.87
	Class D	5,321,320		2.64
Batch 4: 01/04/2024 to 30/04/2024	Class A	0	1,942,263	0
	Class B	200,000		0.10
	Class C	5,325,000		2.74
	Class D	1,693,760		0.87
Batch 1: 01/05/2024 to 31/05/2024	Class A	0	1,798,263	0
	Class B	396,000		0.22
	Class C	5,237,600		2.91
	Class D	2,911,720		1.62
Batch 2: 01/06/2024 to 30/06/2024	Class A	0	1,916,792	0
	Class B	60,000		0.03
	Class C	3,154,000		1.65
	Class D	1,250,400		0.65
Batch 3: 01/07/2024 to 30/07/2024	Class A	0	1,881,592	0
	Class B	290,000		0.15
	Class C	281,000		0.15
	Class D	1,309,800		0.70
Batch 4: 01/08/2024 to 31/08/2024	Class A	0	1,881,592	0
	Class B	0		0
	Class C	424,600		0.23
	Class D	483,800		0.26
Batch 1: 1/09/2024 to 30/09/2024	Class A	0	1,807,410	0
	Class B	140,000		0.08
	Class C	364,600		0.20
	Class D	1,778,560		0.98
Batch 2: 1/10/2024 to 31/10/2024	Class A	0	1,765,200	0
	Class B	54,000		0.03
	Class C	468,600		0.3
	Class D	1,669,000		0.9
Batch 3: 1/11/2024 to 30/11/2024	Class A	0	1,765,200	0
	Class B	72,000		0.04
	Class C	362,000		0.2
	Class D	1,736,480		0.98
Batch 4: 1/12/2024 to 30/12/2024	Class A	0	1,714,280	0
	Class B	15,000		0.009
	Class C	221,220		0.13
	Class D	1,700,360		0.99

## 4 Discussion

This study applied the Animal Health Loss Envelope (AHLE) framework to quantify the economic burden of mastitis and characterize antimicrobial use intensity (AMUI) within a commercial dairy production system. Using 4 years of longitudinal farm management data, the results demonstrate that mastitis represented a persistent and economically important health challenge, generating substantial costs through treatment expenditure, preventive investments, production losses, and animal removal. The study highlights how integrating disease occurrence, management responses, and resource use provides a more comprehensive understanding of livestock disease burden at farm level.

### 4.1 Mastitis as a persistent economic burden

The consistently high mastitis incidence observed across the study period indicates that the disease represented an ongoing production constraint rather than occasional outbreak events. Although recovery rates exceeded 90% in all years, the large number of cases translated into cumulative economic losses, largely driven by repeated treatment events and productivity reductions. This finding aligns with previous studies demonstrating that mastitis imposes significant economic losses even under well-managed dairy systems due to the recurrent nature of the disease and its associated management requirements (1, 2).

Importantly, mortality and mastitis-related culling, while relatively low in proportion to total cases, contributed disproportionately to overall economic losses by removing productive animals from the herd. Within the AHLE framework, these outcomes represent irreversible productivity losses that accumulate over time and influence herd profitability beyond immediate treatment costs.

### 4.2 Management intensity and treatment outcomes

High treatment coverage and recovery rates suggest that the farm employed intensive and effective mastitis management strategies. However, achieving these outcomes required sustained investment in therapeutic interventions and supportive care, highlighting the economic trade-off between disease control and production efficiency. These findings illustrate that successful clinical management does not necessarily equate to reduced economic burden, as maintaining high recovery rates may involve considerable financial input.

The results therefore emphasize that the economic impact of mastitis is shaped not only by disease severity but also by the intensity of management responses required to maintain herd performance. This aligns with livestock health economics perspectives in which disease costs arise from both biological losses and the resources invested in mitigating them.

### 4.3 Antimicrobial use

Patterns of antimicrobial use (AMU) observed on the farm demonstrate a strong dependence on Class C and Class D antimicrobials, which accounted for most of the total milligrams used across batches. This is consistent with typical mastitis management in dairy systems globally, where broad-spectrum intramammary and systemic agents form the backbone of treatment strategies (6). The seasonal distribution of AMU, with pronounced peaks between January–March and October–December, reflects documented seasonal mastitis dynamics driven by humidity, heat stress, and increased pathogen load during wet months. This alignment between disease pressure and antimicrobial consumption highlights the disease-driven nature of AMU, a pattern also reported in other intensive dairy systems in both high- and low-income countries (1).

The AMU intensity (AMUI) values recorded, ranging from 0.03 mg/kg to 4.64 mg/kg, underscore substantial temporal variability in antimicrobial exposure. These values fall within the global range reported for dairy production systems but exceed those documented in countries with advanced mastitis control programmes (15, 16). High AMUI values, especially in early lactation months, correspond to increases in clinical mastitis incidence and the need for repeated treatments after initial therapeutic failure. This suggests the presence of suboptimal therapeutic responses, consistent with treatment-refractory cases described in the literature as indicative of underlying antimicrobial resistance (7, 10).

The farm's minimal or zero use of Class A antimicrobials reflects strong antimicrobial stewardship practices, particularly the prevention of use of critically important antimicrobials for human medicine. This approach aligns with recommendations from the World Health Organization (WHO) and OIE/WOAH, which emphasize limiting Class A agents in food animals to preserve their effectiveness in human healthcare ((11, 12). The consistent avoidance of fluoroquinolones, 3 rd/4 th-generation cephalosporins, and colistin reflects exemplary alignment with global stewardship guidelines.

The pre-dominance of Classes C and D antimicrobials, although justified for mastitis treatment, raises considerations about AMR selection pressure within the farm environment. According to Morel et al., (3), high-volume antimicrobial use, even within non-critical classes, can sustain selective pressure and contribute to the emergence of resistant bacterial populations that later become difficult to treat.

From a farm-level perspective, the AMU patterns highlight how production pressures, veterinary access, farmer decision-making, and environmental conditions interact to shape antimicrobial consumption (2). In months with high biomass production and high milk output, the cost of losing cows to mastitis becomes greater, creating incentives for increased AMU. Cooper and Okello, (13) describe such situations as classic “AMU-AMR trade-off environments,” where the immediate economic need to salvage milk production conflicts with long-term AMR sustainability objectives.

The observed batch-level variability suggests that AMU is responsive to disease incidence, but also influenced by management factors such as calving patterns, environmental hygiene, and feed quality. Bao et al., (14) demonstrated that investments in water, sanitation and hygiene (WASH), hygiene, disinfectants, vaccination and improved cow environment significantly reduce the need for antimicrobials. The current farm's heavy investment in disinfection, vaccination, and selective dry cow therapy likely contributed to lower AMU in several batches, demonstrating the effectiveness of preventive interventions.

Overall, the AMU and AMUI patterns on the farm highlight the complex interaction between disease ecology, management practices, economic incentives, and AMR pressures. Monitoring AMU at a granular, batch-level scale provides insights crucial for designing targeted stewardship interventions. Integrating AMU metrics into AHLE frameworks helps quantify how antimicrobial use contributes to the burden of disease and AMR, supporting evidence-based decision-making for optimizing treatment, prevention, and long-term herd health outcomes.

### 4.4 Antimicrobial use intensity within mastitis management

Antimicrobial use formed a central component of mastitis treatment expenditure, and analysis of antimicrobial use intensity (AMUI) provided additional insight into management practices. Temporal variation in AMUI corresponded with periods of increased mastitis incidence, suggesting that antimicrobial consumption was primarily disease-driven rather than routine or prophylactic. This relationship highlights the value of AMUI as a management indicator within economic analyses, allowing antimicrobial use to be interpreted in relation to disease pressure and treatment demand.

Importantly, the study did not aim to estimate antimicrobial resistance burden or attribute treatment outcomes to resistance mechanisms. Instead, AMUI was used to characterize patterns of antimicrobial consumption within mastitis control strategies, providing a quantitative measure of management intensity that complements economic and epidemiological indicators.

### 4.5 Economic pathways within the AHLE framework

Applying the AHLE framework allowed disease impacts to be interpreted through multiple interconnected pathways. Direct economic losses were driven by expenditures on treatment, diagnostics, and preventive interventions, while indirect losses were associated with discarded milk, reduced productivity, mortality, and culling. The integration of these components demonstrates how persistent disease pressure creates cumulative economic effects through repeated cycles of treatment and production disruption.

This systems-based interpretation is particularly valuable for commercial dairy enterprises, where management decisions must balance productivity goals with resource allocation for disease control. The study illustrates how routine farm-level data can be leveraged to generate meaningful insights into disease burden without requiring complex modeling approaches by combining epidemiological indicators, antimicrobial use metrics, and economic outcomes.

### 4.6 Implications for dairy management and policy

The findings suggest that improving preventive mastitis control measures may offer greater long-term economic benefits than relying primarily on treatment-based management. Strategies aimed at reducing disease incidence, such as enhanced milking hygiene, early detection, and improved herd management practices, could lower both treatment costs and antimicrobial use intensity, thereby improving economic efficiency.

From a broader perspective, the study demonstrates the practical application of the AHLE framework within intensive dairy systems in sub-Saharan Africa, where empirical economic data on livestock disease burden remain limited. Generating such evidence is essential for informing farm-level decision-making and guiding investments in sustainable livestock health management.

### 4.7 Study limitations and future research

Several limitations should be acknowledged. First, the analysis was based on data from a single commercial dairy farm, which may limit the generalisability of findings to other production systems. Second, subclinical mastitis cases were not systematically captured within the farm database, potentially underestimating total disease burden. Third, although antimicrobial use intensity was quantified, the study did not include pathogen-level antimicrobial susceptibility testing, and therefore treatment outcomes cannot be interpreted as evidence of antimicrobial resistance.

Future research should expand this framework to multiple farms and production settings to allow comparative analyses and improved external validity. Integrating more detailed disease monitoring and management data would further strengthen the ability to evaluate economic and antimicrobial use dynamics within livestock systems.

### 4.8 Methodological limitations of the AHLE framework

While the Animal Health Loss Envelope framework provides a structured approach for integrating production losses and health expenditures, several limitations should be acknowledged. First, AHLE outputs are highly dependent on the quality and completeness of farm-level data, which may influence the precision of economic estimates. We selected the farm based on its intensive production and comprehensive data collection and recording systems, hence mitigating the limitation.

Second, the framework is primarily descriptive and does not establish causal relationships between disease and observed productivity changes, meaning that some losses may be influenced by confounding management or environmental factors. In addition, AHLE simplifies complex biological and management interactions, and therefore may not fully capture indirect or long-term consequences of disease.

Finally, although antimicrobial use metrics can be incorporated, the framework does not directly quantify antimicrobial resistance-attributable losses without supporting microbiological evidence. These limitations highlight the importance of cautious interpretation and the need for complementary analytical approaches in future studies.

## 5 Conclusion

This study applied the Animal Health Loss Envelope (AHLE) framework to quantify the economic impact of mastitis in a commercial dairy farm using 4 years of routinely collected production and health management data. The findings demonstrate that mastitis represents a substantial and recurrent economic burden, driven by high disease incidence, continuous treatment requirements, and significant productivity losses. Although recovery rates were consistently high, the cumulative effects of treatment expenditures, preventive investments, discarded milk, mortality, and mastitis-related culling resulted in considerable financial impact at herd level.

The analysis highlights that the economic burden of mastitis is shaped not only by disease occurrence but also by the management intensity required to sustain production under intensive dairy systems. Antimicrobial use formed an important component of disease control expenditure, reflecting its role within mastitis management strategies, while preventive investments in hygiene, vaccination, and biosecurity contributed substantially to overall health-related costs.

This study provides empirical evidence of how routine farm-level data can be used to estimate livestock disease burden in real production settings by integrating disease occurrence, production losses, and health expenditures within the AHLE framework. The findings emphasize the importance of strengthening preventive mastitis control strategies to reduce recurrent losses and improve economic efficiency within dairy enterprises.

Future research should expand this approach across multiple farms and production systems to improve generalisability and to support comparative assessments of disease management strategies. Integrating herd-level economic evaluations with enhanced monitoring of antimicrobial use and disease outcomes will further support evidence-based decision-making for sustainable dairy production.

## Data Availability

The original contributions presented in the study are included in the article/supplementary material, further inquiries can be directed to the corresponding author.
